# Skywalking in the city: Glass platforms and the architecture of vertigo

**DOI:** 10.1016/j.emospa.2017.05.005

**Published:** 2018-08

**Authors:** Davide Deriu

**Affiliations:** Faculty of Architecture and the Built Environment, University of Westminster, 35, Marylebone Road, London NW1 5LS, UK

## Abstract

• The paper explores the ambivalent concept of vertigo and its significance for contemporary architecture.
• It examines in particular the rise of elevated glass platforms through concepts of transparency, experience, and kinaesthesia.
• Proposes that these emerging design features constitute a kind of ‘sixth façade’.
• Discusses this phenomenon as a spatial manifestation of the experience economy.
• Concludes by highlighting the rise of ‘architectures of vertigo’ in relation to wider social imperatives.

## 1 Introduction

Ever since the advent of high-rise architecture, in the late nineteenth century, the modern city has been a distinct locus of vertiginous experience. Whilst the correlation between vertigo and tall buildings might at first appear to be an obvious one, it is in fact a variable function of ever-evolving techniques and materials, as well as depending on the psychosocial conditions that underlie the experience of space at a given place and time. A great deal of public interest has been aroused over the past decade by the proliferation of glass-floored viewing platforms, which have become increasingly popular features of observation decks around the world. These platforms, often branded as ‘dare to walk on air’ experiences, are designed to challenge the user's perception of spatial depth. Whilst older types of viewing galleries, such as open decks with low walls, could provoke stronger feelings of height vertigo than new glass floors (not least because of the imagined agency of throwing oneself off the high point), the latter are distinct insofar as they are designed to conjure the thrill of walking over the abyss in a seeming state of suspension.

The rise of glass floors gathered momentum in the mid-noughties, at a time of rapid growth of vertical cities (King, 2004) that saw a new wave of ‘supertall’ and ‘megatall’ buildings emerge around the world. Social implications of contemporary high-rise construction have been investigated from various perspectives, with regard to the vertical dimension of cities (Graham and Hewitt, 2012, Graham, 2016); the role of the skyscraper in architectural culture (Nobel, 2015) and in tourism-led urban regeneration (Leiper and Park, 2010); and the psychological influence of tall buildings on their occupants (Gifford, 2007). Meanwhile, the vertical visualisation of space brought about by the combined use of satellite imagery and digital technologies, such as Google Earth (Di Palma, 2008), has affected the conditions of embodied seeing as well as the physical experience of vertigo, ushering in a new ‘age of aerial vision’ (Gilbert, 2010). The recent surge of thrill-seeking practices such as *rooftopping*, which has sparked a broad diffusion of ‘vertigo inducing’ images on the web, is further signal of a wider shift in urban experience and representation (Deriu, 2016). Seen together, these phenomena are symptomatic of a wider socio-cultural condition that appears to be pervaded by a dizzying spatiality, particularly acute in vertical cities.

The term vertigo crops up in architectural and urban discourse rather frequently, albeit mainly in a figurative sense. A case in point is the eponymous Glasgow exhibition (1999), where ‘The Strange New World of the Contemporary City’ was illustrated through an assortment of architectural projects that ranged widely in function and scale – from Tate Modern in London to the Ontario Mills shopping mall in California. In the exhibition volume, Tate Modern's architect Jacques Herzog remarked:

‘The word “vertigo” does not have auspicious connotations. In fact, it would seem to address the sinister and even dangerous side of things: fear of heights and the attendant dizziness. Or even a double anxiety: the fear of falling passively through no fault of one's own, and the fear of responding quasi-actively to the magical attraction of the abyss and thereby succumbing to its vertiginous appeal. “Vertigo” could be said to express an inescapable ambivalence and indeterminacy.’ (Herzog, 1999: 6)

The show did not have a specific agenda, nor did it claim to present a consistent design approach. Instead, the curatorial strategy aimed to capture the generic state of ‘dizziness’ and ‘disquiet’ provoked by the global architectural landscapes of the 1990s (Moore, 1999). The provocative, and somewhat prophetic, title echoed the spiralling tension that runs through Alfred Hitchcock's *Vertigo*: an enduring point of reference for cinematic representations of the city as a protean emotional landscape.

**Fig. 1:**
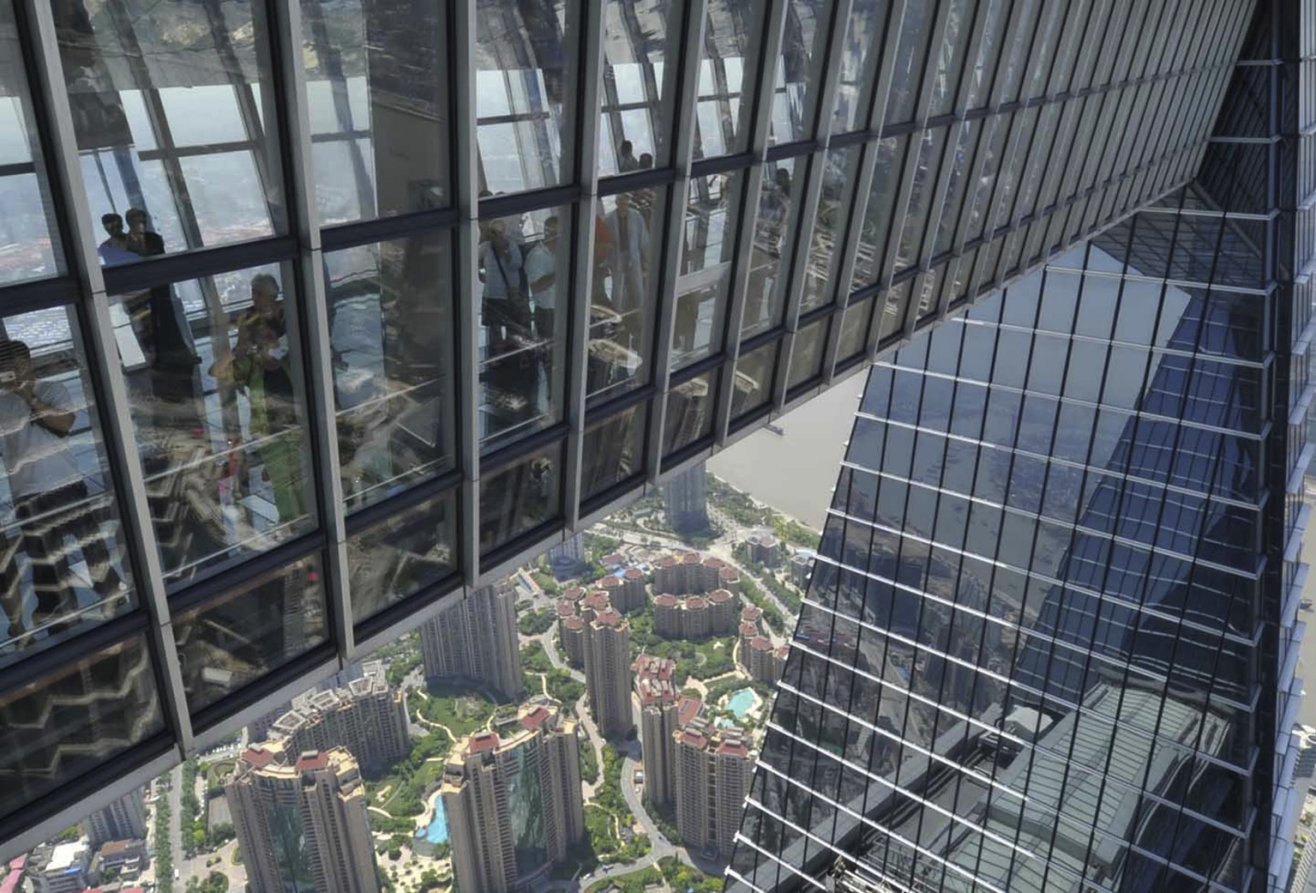
Shanghai World Financial Centre. View of the 'Skywalk 100′ observatory. Source: SWFC Observatory (http://swfc-shanghai.com/).

Two decades on, the ‘strange new world’ portrayed on the eve of the Millennium appears all too familiar. Indeed, the ‘double anxiety’ evoked by Herzog has meanwhile taken up a new dimension. Today, vertigo aptly describes the physical sensation that is induced by architectural elements such as the fashionable glass-floors that line many high-level walkways. This vogue is epitomised by the Shanghai World Financial Centre (SWFC), which was featured in the Glasgow exhibition when only the foundations had been laid. Its construction was eventually completed in 2008 following design alterations that increased the overall height of the tower to 492 m. The SWFC tower contains an exemplar of the current trend for immersive viewing experiences: besides panoramic vistas of the surrounding cityscape, the 100^th^-floor observation gallery, situated at 474 m of height, offers vertical views through a series of transparent glass panes built into the floor (see Fig. 1). The SWFC has since boasted ‘the world's highest observatory’, a record that may not last for long as the global race to the sky carries on unabated.

The construction of similar design features around the urbanised world suggests that architectural vertigo has become a sought-after phenomenon. This trend raises questions about the conditions in which space is designed, perceived and experienced in the contemporary city. What bodily experiences are implicated in the ‘states of suspension’ induced by these platforms? What are their material and spatial properties? And how do these spaces relate to the wider socio-economic context in which they are produced? A cross-disciplinary approach will inform the investigation of these issues, while a series of design projects will serve to illustrate various manifestations of the subject. The proposed interpretation draws on theories of transparency and sensory experience of space, supported by insights from psychology, and ultimately critiques high-level glass platforms as ‘tourist bubbles’ that crystallise, quite literally, a social imperative of the present moment.

## 2 Vertigo in the city: architecture and *ilinx*

The term vertigo is fraught with multiple and ambivalent meanings that cannot be exhausted in an article. Some elucidation may nonetheless inform a critique of current architectural trends. Whilst in medical discourse vertigo is usually treated as a symptom of balance system disorders, in popular culture the word is used more loosely to evoke various sensations of giddiness, dizziness, and disorientation that are associated with a perceived loss of equilibrium. Dictionary definitions range widely, from the illusion of physical movement (‘the act of whirling round and round’) to the bodily perception related to it (‘swimming in the head’), and extend to figurative meanings (‘a disordered state of mind, or of things, comparable to giddiness’) (OED). In a figurative sense, the term has also been adopted to describe the precarious conditions of life in contemporary societies. For Young (2007: 12), ‘Vertigo is the malaise of late modernity: a sense of insecurity of insubstantiality, and of uncertainty, a whiff of chaos and a fear of falling.’ Accordingly, a generalised feeling of giddiness defines our ‘liquid’ modernity (Bauman, 2000), an epoch in which values that previously had a solid foundation, such as social status and economic position, have become increasingly fluid and unstable.

The notion of ‘groundlessness’ has gained currency in art, architectural, and urban discourses over the past decade (Dorrian, 2009, Steyerl, 2011, Graham, 2016). Dorrian (2009) in particular draws comparisons between the ‘dissolution’ of ground evoked by Hitchcock, as well as by authors like Nabokov and Sebald, and the disorienting experience induced by contemporary architectures such as London's City Hall. At the bottom level of this Foster-designed building, visitors can stand on a giant aerial photomap of London, taking symbolic possession of the city from a vantage point that traditionally signifies a position of power and control. Dorrian (2009: 86) sees this as ‘an attempt to architecturally stage […] democratic transparency’, in a similar mould as the glass dome of the new Reichstag in Berlin – designed by the same architect. At City Hall, the abstract miniaturization of London produced by the aerial view somehow jars with the act of walking on the photomap while looking down in search of familiar clues. If, on the one hand, this embodied experience provides the visitor with a sense of grounding, on the other hand the photomap triggers a ‘vertiginous multiplicity’ that causes an opposite ‘ungrounding’ effect: a disorientation amplified by the non-hierarchical code of representation that distinguishes satellite imagery from cartographic maps (Dorrian, 2009: 91).

The ‘vertiginous ungrounding’ described by Dorrian calls to mind philosophical conceptions of modernity as an existential condition perturbed by unprecedented degrees of freedom: namely, the ‘dizziness of freedom’ expounded by Kierkegaard in his 1844 treatise *The Concept of Anxiety* (LeBlanc, 2011). However, while Dorrian plays down the role of heights in the etymology of vertigo and its aforementioned cultural representations, there is evidence to suggest that elevation is in fact increasingly bound up with the dizzying experience of contemporary urban space. As we shall see, the current popularity of elevated glass platforms vividly illustrates how height vertigo is elicited in visceral ways through particular spatial arrangements.

Remarkably, the notion of vertigo is conspicuous for its absence from the main texts that have defined the critical discourse on the perception of the city from above. Barthes (1983/1964) omitted it from his ‘Eiffel Tower’ essay, wherein he coined the term ‘architectures of vision’ (*architectures de la vue*) to describe the 19th-century structures that turned the ‘fantasy of a panoramic vision’ into material reality. Accordingly, the Tower epitomised the rise of a modern form of perception that made it possible to embrace a bird's eye view of the metropolis and thereby to comprehend it in its structure. By positing the city as a text to be read and deciphered, Barthes's argument privileged the power of intellection over the corporeal experience of space. A similar tendency to reduce the observation tower to a mere vantage point characterises De Certeau's (1984/1980) later description of Manhattan from atop the World Trade Center, wherein the ‘voluptuous pleasure’ of seeing the city as a whole is described as a purely visual act. The observation deck is the platform from which a *dieu voyeur* observes the city ‘like a picture’ and lays claim over it. The ensuing ‘fiction of knowledge’, concludes De Certeau (1984/1980: 92), ‘is related to this lust to be a viewpoint and nothing more.’

While these now-classic accounts remain critical for our understanding of urban modernity, and of the observation deck as one of its enduring topoi, they leave unquestioned the embodied experience of space that was induced by such unprecedented heights. How does one *feel* when confronting the city from high vantage points? This is not to diminish the cognitive function of viewing galleries, nor to dispute their significance as social spaces implicated in procedures of spectacle and surveillance; but rather to address another dimension of high-rise architecture that has become all the more relevant in light of the current vogue of glass floors. The design of platforms that expand the field of vision to the vertical axis affects the practice of viewing cities from above in ways that have not been interrogated as yet. In order to comprehend this phenomenon, let us turn to another theory that puts a different spin on the notion of vertigo.

In his seminal book, *Man, Play and Games*, Caillois (2001/1958) revived the ancient Greek word *ilinx* (‘whirlpool’) to define one of the four fundamental categories of human play. With this term Caillois identified those games ‘which are based on the pursuit of vertigo and which consist of an attempt to momentarily destroy the stability of perception and inflict a kind of voluptuous panic upon an otherwise lucid mind.’ (Caillois, 2001/1958: 23) He remarked that *ilinx* is an ancient form of play, as exemplified by the rituals performed by whirling dervishes and pole-flying *voladores*. In the course of history, fairgrounds became traditional spaces for games of vertigo, with their ‘machines for rotation, oscillation, suspension, and falling, constructed for the sole purpose of provoking visceral panic.’ (Caillois, 2001/1958: 133) Crucially, Caillois observed that *ilinx* was a pervasive aspect of modern life, typified by the exhilaration induced, for instance, by speed and drugs.

Historically, the quest of ‘voluptuous panic’ described by Caillois found new expression in the 19th century through a range of mechanised thrill rides epitomised by the rollercoaster. While gravity plays had long been popular social entertainments in funfairs and circuses, the amusement park turned the modern city into a playground for thrilling pleasures (Kane, 2013). These ‘mechanised entertainments’ emerged at a time when a series of bodily practices aimed at defying the force of gravity – such as high diving, parachuting, and funambulism – became mass spectacles. In this respect, the 19th century has aptly been called ‘the gravity century’ (Soden, 2003). It is interesting to note that Caillois singled out tightrope walking as the activity that most closely corresponds to *ilinx*: by turning the ludic – and, therefore, unproductive – nature of play into a performance art, the wire-walker ‘[moves] through space as if the void were not fascinating, and as if no danger were involved.’ (Caillois, 2001/1958: 137). This practice, which burgeoned as a public spectacle in the 1850s, over the twentieth century moved from the abysses of nature to those of cities. In recent decades, skillful acrobats like Philippe Petit and Nik Wallenda have displayed their ability to master vertigo in the midst of breathtaking urban environments (Johnston, 2013).

Caillois's anthropology of play has inspired urbanists to engage with the ludic aspects of contemporary cities. Stevens (2007: 43), for instance, draws extensively on the concept of *ilinx* in an attempt to unearth the creative potential of public spaces: ‘[v]ertigo negates instrumental benefit and embraces risk for its own sake and the affirmation of human bodily experience.’ From a different angle, Caillois's theory might also inform the critical analysis of high-level glass platforms as stages where games of *ilinx* are socially performed. For the challenge of ‘walking on air’ in some way simulates the acrobatics of highwire walkers who confront the vertiginous heights of cities. As shown in the next section, the vogue of glass floors makes the thrill of ‘skywalking’ accessible to the general public within safe and protected environments – usually for the price of a ticket.

## 3 Augmenting architecture: the rise of glass floors

The use of glass in the design of walking surfaces is not new, and has a particularly rich background in retail interiors. Notably, the dramatic glass staircases designed by Eva Jiřičná from the 1980s onwards paved the way for a trend that, over the past decade, has been spread globally by the Apple store chain. In the noughties, while translucent glass became a popular means of allowing sunlight through multi-storey spaces, the design of transparent glass floors also gained momentum. A typical use can be found in exhibition spaces such as archeological sites and museums, where visitors are drawn to contemplate objects on display underneath their feet. Although transparent glass panes are usually placed over shallow spaces, there are exceptions that engage a bodily interaction with deeper voids. At the Acropolis Museum in Athens, for instance, structural glass was used extensively not only to exhibit the outdoors archeological excavations but also to create elevated, see-through surfaces that allow users to walk over the museum's central space (Self, 2014).

**Fig. 2:**
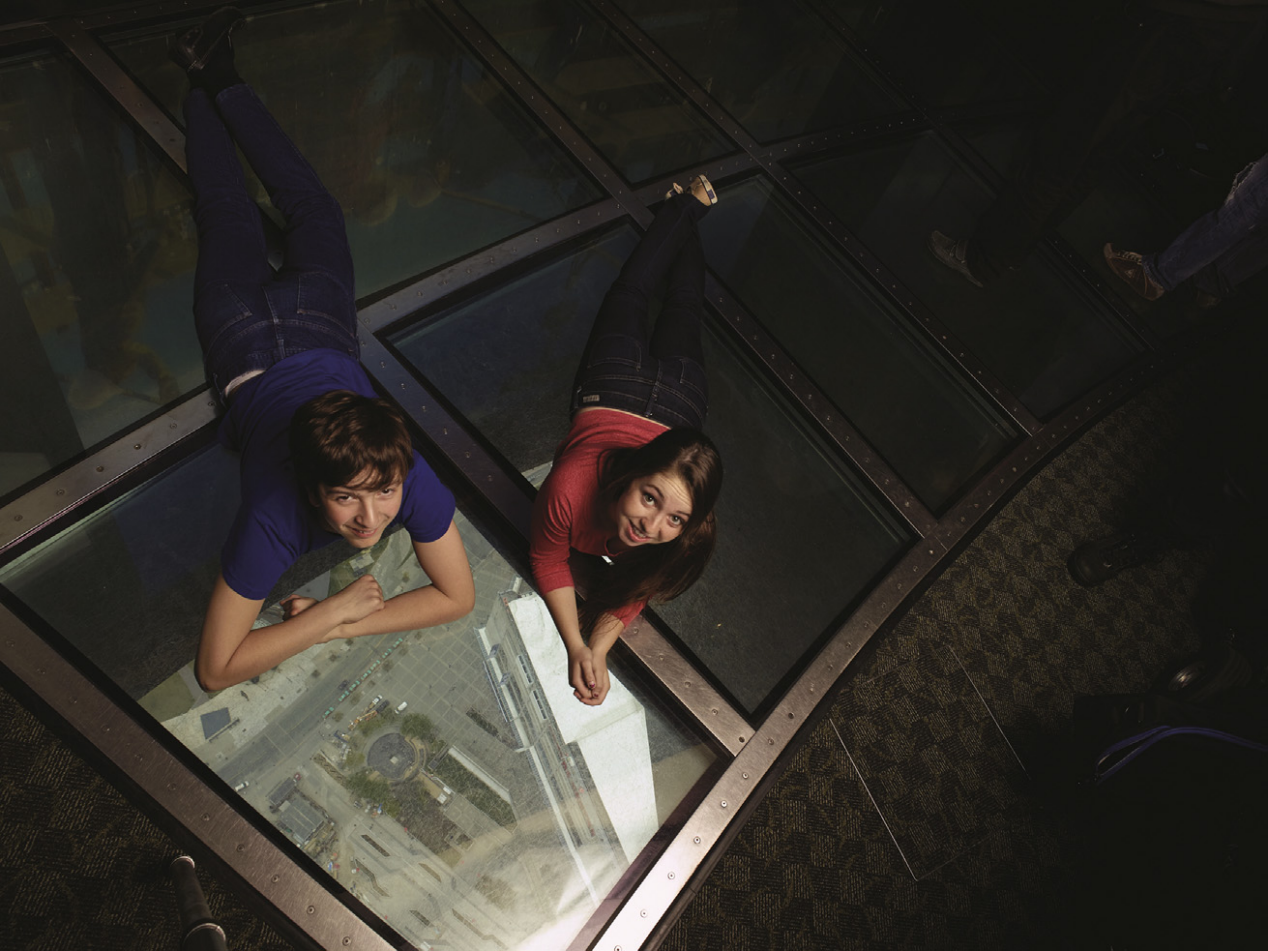
Glass floor at the CN Tower, Toronto. Copyrights: Canada Lands Company CLC Limited.

Different considerations preside over the design of glass-bottomed platforms in tall structures, where the main purpose is to open up downward-looking perspectives onto the yawning void underneath. The first high-level transparent floor was fitted in the observation deck of Toronto's CN Tower, where in 1994 a horizontal grid of tempered glass panes was mounted on the viewing gallery 342 m above the ground (see Fig. 2). After a few early emulations, this project set a worldwide trend only a decade later. The CN Tower boasted the highest glass-floored observation deck in the world until 2008, when it was overtaken by the Shanghai World Financial Center (SWFC): by that time, the global craze for skywalks had become pervasive.

Whilst the CN Tower heralded the glass floor as a means of enhancing the tourist appeal of high-level viewing galleries, the SWFC Observatory embodies its evolution to a full-fledged spatial concept. Located in the midst of Shanghai's financial district, the skyscraper symbolizes the boom of high-rise construction that took place in China amidst major changes in the global economic geography around the turn of the 21st century. Glass floors furnish the walkways that span the 55 m-long gallery on the tower's 100^th^ floor – a transparent bridge suspended nearly half a kilometer above the ground. The ‘Skywalk 100’ epitomises a new type of urban observatory: a free-standing gallery that envelops the visitor's body within a glassy capsule-like space from which they can watch the city from above while enjoying the thrill of ‘walking on air’. The dizzying quality of this space is further heightened by the reflecting ceilings and the outward inclination of the fully-glazed side walls. As the SWFC website proclaims: ‘Walking on the three transparent glass-walled walkways, visitors will experience the feeling that Shanghai lies at their feet.’ (SWFC) Although the possibility of gazing at the city is still very much part of the attraction, the skywalk signals a move from Barthes's architectures of vision towards what might be called ‘architectures of vertigo’. No longer a pure machine for seeing, the observation deck is reconfigured as a machine for thrilling: a spatial enclosure that combines the visual spectacle of the city with an altogether more visceral experience.

**Fig. 3:**
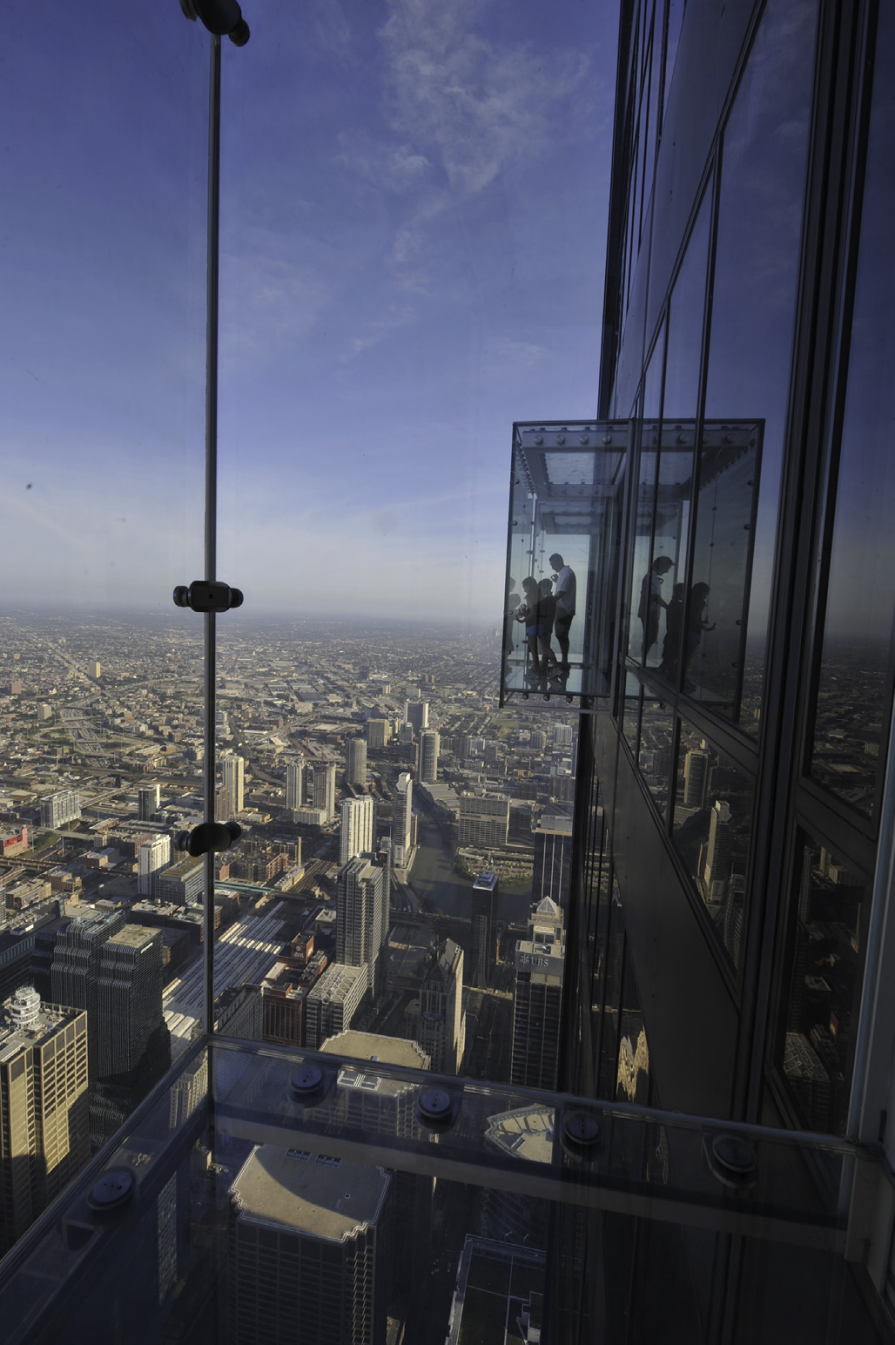
‘The Ledge’, Willis Tower, Chicago. Copyrights: Skydeck Chicago at Willis Tower.

Over the past decade, glass floors have become recurring features of viewing platforms around the world.^1^ Two notable examples are located in Chicago, the historical birthplace of both the skyscraper and the observation wheel. In 2009 the Willis Tower (formerly Sears Tower) had its 103^rd^-floor Skydeck retrofitted with a series of retractable balconies designed by SOM – the firm behind the original 1970s tower. This feature, dubbed ‘The Ledge’, broke new ground in the design of high-altitude viewing platforms owing to its four all-glass boxes that protrude over 1.3 m from the building façade at 412 m above ground (see Fig. 3). The challenge to step on a fully transparent balcony at such dizzying heights is advertised as a brave act: ‘Get out on the Ledge – *if you dare*’ (Skydeck Chicago). Those who dare shall be rewarded: ‘With glass on the ceiling, floor, and all sides, it is truly, an unforgettable experience.’ (ibid.) Behind the platitude of this marketing pledge lies a deeper economic strategy that pits observation towers vying for tourists' attention against each other – globally as well as locally.

**Fig. 4:**
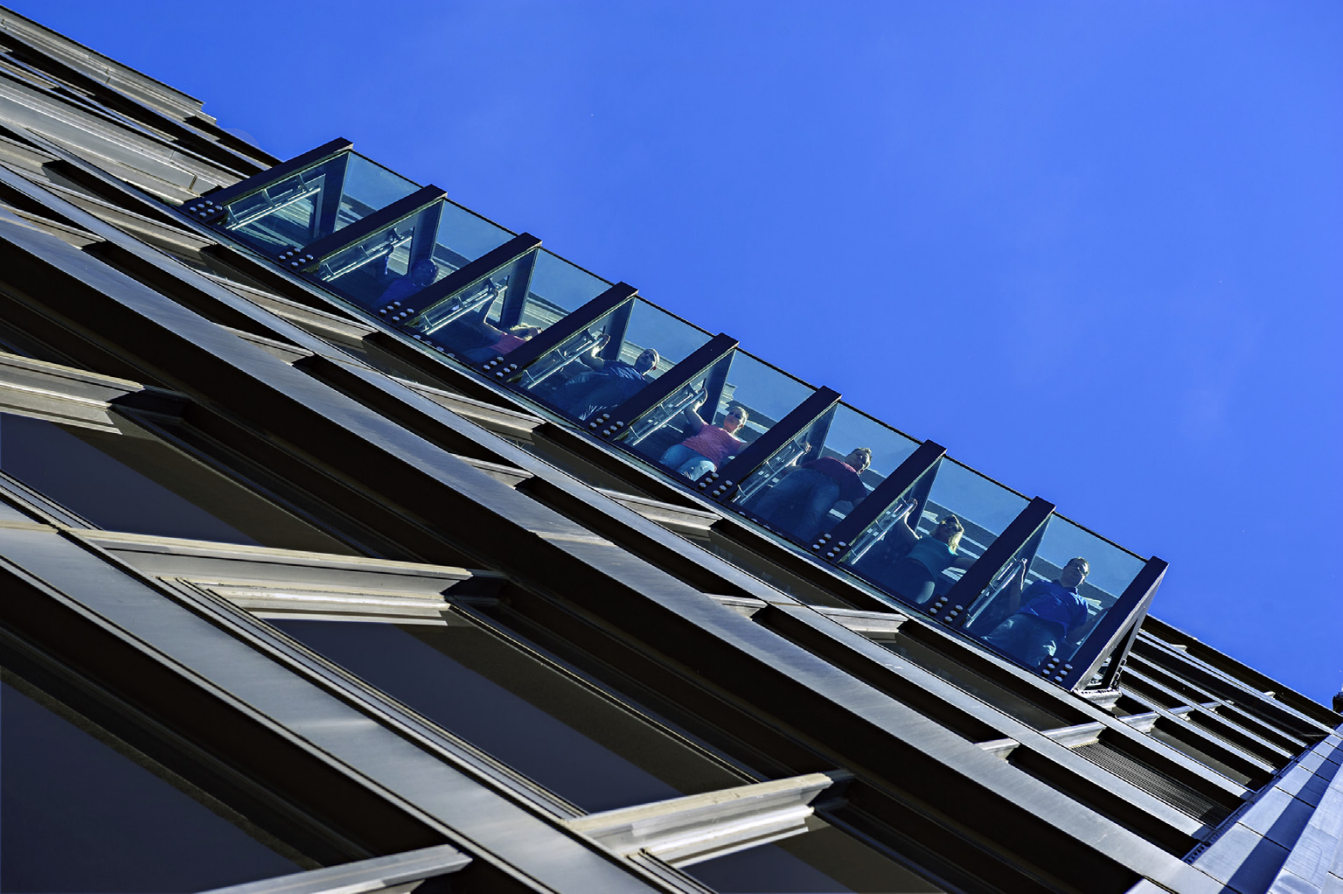
‘Tilt’, John Hancock Center, Chicago. Copyrights: 360 Chicago.

In 2014 a new attraction called ‘Tilt’ opened across town at the former Observatory of the John Hancock Center, in overt competition with Skydeck Chicago. The feature consists of a glass-and-steel platform that tilts thirty degrees outwards, allowing visitors to lean with their bodies over a 300 m-chasm (see Fig. 4). The conception of this immersive space, branded ‘Chicago's Highest Moving Experience’ (360 Chicago), signalled that even one of the highest viewing galleries in the world, capable of 360-degree views of the cityscape, needed a facelift to keep up with the competitors. The Tilt presents an interesting variation on the theme of high-level transparent features: an alternate tendency towards the design of moveable elements aimed at stimulating a dynamic, vertigo-inducing experience. The game of architectural *ilinx* here requires that you let yourself go, quite literally: transported over the void, the visitor-cum-player enjoys the adrenaline rush provoked by the momentary disruption of sensory stability.

The dynamics of thrill-seeking have been taken to a new extreme at the U.S. Bank Tower in Los Angeles, where an entirely glass-cladded attraction called ‘Skyslide’ was built in 2016 as an extension of the ‘Skyspace’ observation deck. Hanging on the building's façade at circa 300 m of height, the Skyslide entices visitors to ‘experience […] unparalleled views in a whole new way as they glide from the 70^th^ to the 69^th^ floor of the U.S. Bank Tower.’ (OUE Skyspace LA) Local commentators have noted that the Skyspace project aimed to bring tourism revenue to an area of Downtown L.A. with rising vacancy rates in commercial skyscrapers (Hawthorne, 2016). The costly makeover of the tower's observatory has been regarded as an attempt to reclaim, and reenchant, the view from above in a sprawling city where the aerial gaze is mainly associated with the helicopter view (ibid.). The Skyslide marks a further step in the design of high-altitude glass boxes. This transparent device induces the ‘voluptuous panic’ of a free fall into space: a fast yet safe descent that derives its allure, yet again, from the attraction of the void.

Meanwhile, a parallel development has taken place in Europe, where glass platforms have become familiar attributes of tall structures – albeit at a more modest scale. In the U.K. the craze for glass floors has pervaded new-build and historical architectures alike. Portsmouth's Spinnaker Tower, whose sail-like shape echoes the Burj Al Arab hotel in Dubai, was completed in 2005 to spearhead the regeneration of the harbour area: the glass skywalk that was built on one of the viewing decks (100 m) is deemed to have played a part in the tower's touristic success. More recently, some of the country's major attractions have also been retrofitted with similar features. Blackpool Tower's ‘Walk of Faith’, initially built in the late-1990s as a simple glass-floor panel, was later expanded into a whole skywalk (116 m) as part of the refurbisment unveiled in 2011 by the ‘global leisure’ giant Merlin Entertainments.

**Fig. 5:**
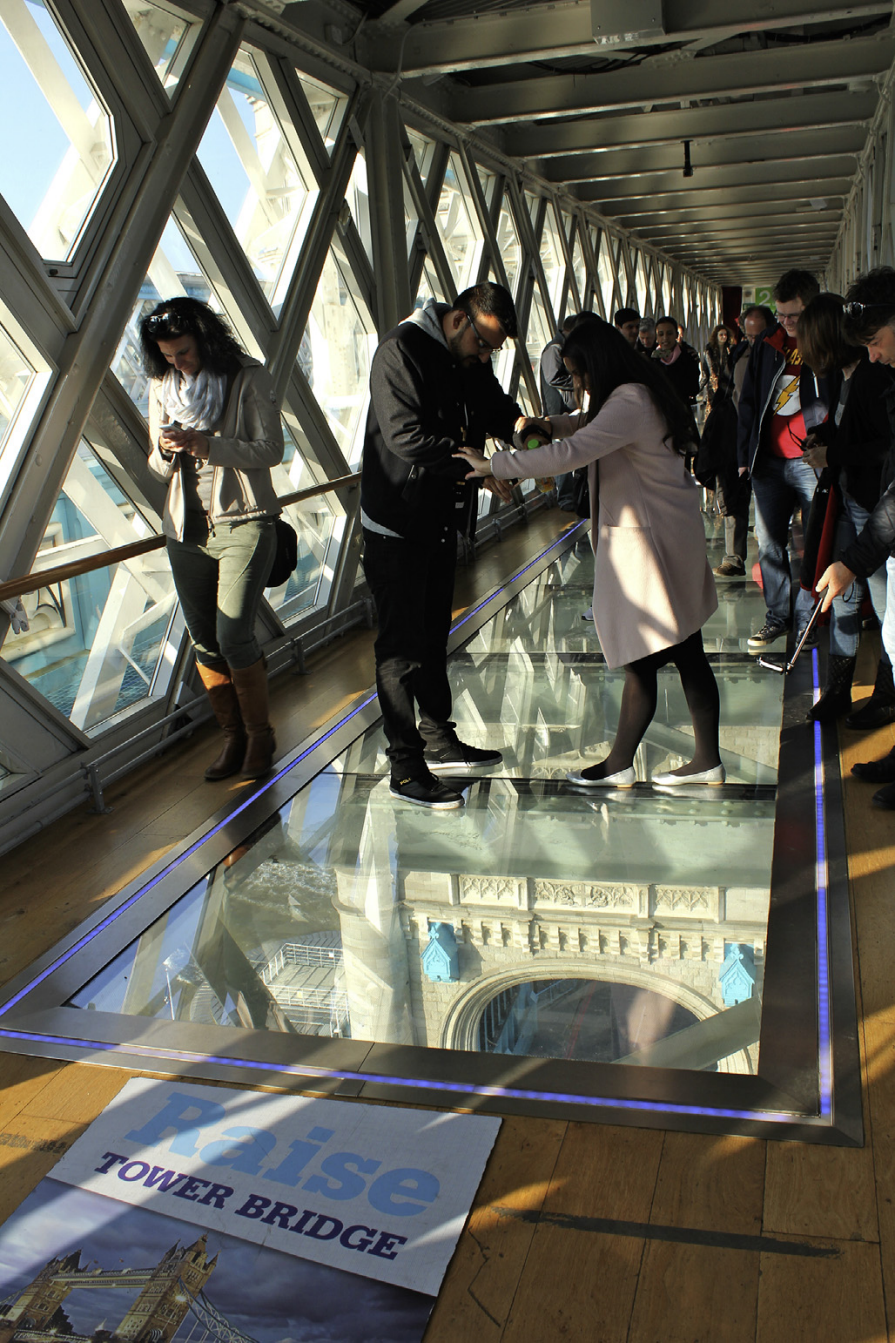
Walkway, Tower Bridge, London. Photo by the author.

Less dramatic in terms of height, yet more interesting in other respects, is the 2014 retrofitting of London's Tower Bridge with glass floors on the panoramic walkways that connect the towers. While the Grade I-listed structure had previously undergone functional renovations, the insertion of 11 m-long, see-through platforms responded to the intent of attracting more users. The revamped twin galleries double up as venues for hire: visitors attending corporate events, private parties, or yoga classes can now revel in – or recoil from – the act of skywalking over the Thames. The relatively limited height (42 m), if compared with other such walkways, allows for a closer visual connection with the passersby and the traffic unfolding on the road bridge down below. The incongruous effect of transparency triggers, in people either side of the walkways, not only sheer curiosity but also an awareness of the reciprocal distances and positions within the newly-opened visual field (see Fig. 5).

**Fig. 6:**
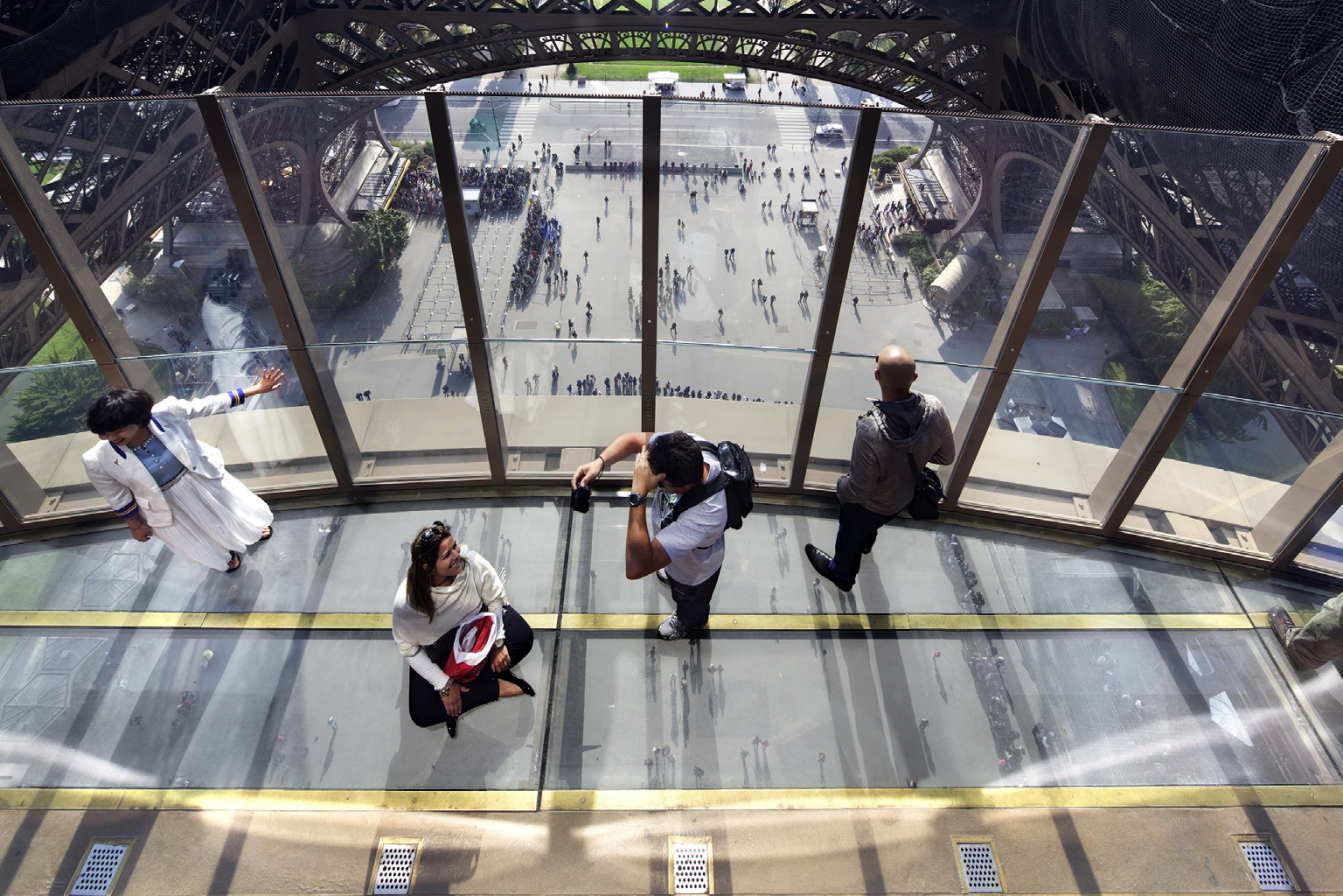
Glass floor, Eiffel Tower, Paris. Copyrights: SETE – Michel Denanc.

A similar intervention took place, around the same time, in the modern ‘architecture of vision’ *par excellence* – the Eiffel Tower. In their restyling of the Tower's first floor (57 m) architects Moatti-Rivière designed a series of glass platforms that aim to achieve an ‘augmented architecture’ (Trétiack, 2014). Besides improving the existing public spaces, the refurbishment included open-air, inward-looking terraces that open up the central void through a combination of horizontal and vertical transparency: ‘The project offers an improved experience of the Tower and Paris, an entertaining sensory experience, a journey of the senses and knowledge.’ (Eiffel Tower, n. d.) In a *Living Architectures* documentary, Alain Moatti explains the idea of magnifying the feeling of void that, historically, has been the primary characteristic of the Tower: a peerless monument which, with reference to Barthes, the architect credits with ‘the invention of third dimension in the city.’ (Bêka and Lemoine, 2014) In a telling scene, a visitor likens the sense of lightness he felt on a glass floor to levitation. The impression of floating on air is further enhanced by the physical contiguity between the horizontal surfaces and the inclined glass parapets that delimit the floor space, which are wholly transparent (see Fig. 6).

Whilst at Tower Bridge the glass walkways were the main raison d’être of the renovation, in the Eiffel Tower they are comparatively minor features of a wider project. The restyling of the Tower is nonetheless symptomatic of a widespread tendency to produce ever-more immersive experiences of space. High-level platforms that challenge users to ‘walk on air’ manifest a pursuit of architectural *ilinx* – a contemporary version of the ‘voluptuous panic’ described by Caillois.

These design features have become common not only in urban settings but also in natural environments, as shown by several projects aimed at heightening the scenic effects of landscapes around the world. The prime example is the cantilevered glass skywalk at Grand Canyon West, Arizona, opened in 2007 and reportedly inspired by Toronto's CN Tower.^2^ Vertiginous structures of this kind play a significant part in the promotion of landscapes that are branded as tourist attractions. As Porter (2015: 207) notes, they signal ‘a tourist development which, like the quest for the tallest building, is a never-ending exercise in one-upmanship, leading further and further into exaggeration.’ The glass floors that have concurrently appeared in observation towers provide an urban analog of the experience of natural landscapes. Indeed, the fact that these platforms were first introduced in cities and only later in natural landscapes is an interesting sign of how the urban environment, with its built-up gorges and canyons, has become a major source of attractions for thrill tourists. In our urban age, in which verticality is an increasingly common dimension of city life (Graham, 2016), architecture has become instrumental to the production of spatialised games of *ilinx.*

## 4 Thrills of transparency: on the ‘sixth façade’

The notion of transparency is key to understanding the spatiality of glass-floored urban observatories. As Forty (2000: 286) notes, transparency is ‘a wholly modernist term, unknown in architecture before the twentieth century.’ Its basic sense, ‘meaning pervious to light, allowing one to see into or through a building’ (ibid.) became popular in the 1910s-1920s, when advances in glass manufacturing and frame construction made it possible to build self-standing glass enclosures. The coupling of structural frame with glass panes, which had an ancestor in the stained-glass windows of Gothic cathedrals, was such a breakthrough in modern construction that has been described as ‘the most significant development in architecture in the last millennium.’ (Fierro, 2003: viii-ix).

A manifest expression of this shift was the curtain wall, which gained broad diffusion after World War II amidst a steep increase in structural glass production. It was in reaction to that trend that Rowe and Slutzky (1963) elaborated the concept of *phenomenal* transparency, a perceptual quality defined as the ‘illusion of spatial depth’ that characterised modernist architecture as well as avant-garde painting. This theory signalled an attempt to transcend the *literal* transparency of the International Style: that is, the basic optical quality of the modernist ‘glass box’. Rowe and Slutzky's sophisticated argument could do little, however, to halt the proliferation of curtain-walls worldwide.

The architectural uses of glass have vastly expanded over recent decades, as this versatile material proved suitable to an increasing number of functions while also providing a symbol of political power (Fierro, 2003). By the turn of the 21st century, with ever more tower blocks dotting the skylines of cities, and the curtain wall defining a global aesthetic (Elkadi, 2006), literal transparency had become so widespread as to conquer the horizontal dimension. And yet, despite their growing popularity, transparent floors are barely mentioned in the literature about glass in architecture (Cruz, 2013). This is all the more remarkable if we consider the novelty of glass platforms that transpose the modernist frame construction onto the horizontal plane. The alteration of the floor into a see-through surface, akin to a horizontal window, calls to mind the notion of ‘fifth façade’ formulated by Le Corbusier in the late 1920s – when he envisioned new functions for the flat roof terrace after flying over South American cities. It might be argued that, by overturning the vertical window-wall onto a horizontal surface, the introduction of the glass floor has ushered in the *sixth façade.* This epithet befits the lower side of elevated buildings and overhanging building elements, insofar as they fulfil the condition of externality that is implied by the physiognomic etymology of the word ‘façade’: that is, in the case of glass platforms, the possibility of looking through from without as well as from within.

For the purpose of the present argument it should be useful to distinguish, with some degree of approximation, between ‘low-level’ and ‘high-level’ glass platforms. The former, such as those at Tower Bridge and the Eiffel Tower, are usually installed in, or added to, the underside of existing structures and effectively operate as horizontal floor-windows that enable a two-way visual contact between inside and outside. Although the main goal of these platforms is to allow for a top-down vision, a bottom-up gaze from below is also afforded, to varying degrees, by the relatively low elevation. Higher platforms hanging several hundred meters above the ground, such as those at the SWFC Observatory and Skydeck Chicago, may not be clearly visible from the street but can nonetheless be seen from within the same buildings as well as adjacent ones. Moreover, the floors' undersides gain wide exposure through media representations, such as brochures and websites, where lower viewing angles are favoured by photographers to visualise the skywalkers' bodies.

The recognition of the field of visibility that is opened up by these transparent surfaces prompts further questions about the spaces they define and the actual uses they enable. What kinds of responses and interactions are elicited by the sixth façade? A complex reciprocal relationship is established between internal users and external viewers. The simple fact that these platforms must remain tightly-sealed in order to perform their function means that visual contact through the glass occurs in a state of spatial separation. The bodies of those who ‘dare to walk on air’ are usually visible from below through a peculiar honeypot effect, as skywalkers perform their balancing acts in front of curious spectactors on both sides of the glass stage. There are nuanced interconnections between how people feel in these spaces and how the latter affect their emotions, since height vertigo manifests itself through a wide range of psycho-physiological responses. Depending of where one sits in the spectrum of ‘height tolerance’ (Salassa and Zapala, 2009), varying degrees of thrill or anxiety can be elicited by states of suspension that appear to defy the laws of gravity.

## 5 Sensing gravity: kinaesthesia at heights

While skywalks are built in highly controlled and safe environments, they are designed to challenge the user's fear of heights by exposing them to the view lying underneath their feet. The structure encasing the glass panes, usually made of multiple-layer tempered glass laid out in a grid, doubles up as a comfort zone where hesitant users can reach for a sense of safety. Those who initially skirt around a glass platform often approach it through small and tentative steps as they confront the fear of the void. This experience transcends the visual field insofar as the act of ‘skywalking’ engages the subject's proprioceptive system as well as the optokinetic one – two distinct perceptual apparatuses whose signals to the brain, if discrepant, may trigger a sensation of dizziness (Yardley, 1994). In fact, the sensory stimulation caused by glass platforms is not confined to the five senses but rouses a sixth sense traditionally known as *kinaesthesia,* or the ‘muscle sense’. As Çelik (2006) points out, this sense was discovered in the early 19th century by psychologists who realised that muscles are capable of receiving sensations from the spinal cord and should therefore be considered to be sentient:

‘Kinaesthesia, the sense of bodily movement […] [refers] to those unclassifiable sensations that could not be traced accurately to one of the five known sense organs, but seemed to originate from the undifferentiated mass of the viscera.’ (Çelik, 2006: 159).

Although the development of neuroscience has greatly expanded the scientific knowledge of body-mind relations, the notion of kinaesthesia remains relevant to the present discussion. It resonates with the prevailing conception of architecture as a field of multi-sensory experience, which, especially since the 1990s, has been largely informed by phenomenology (Holl et al., 2006). Pallasmaa, 2006, Pallasmaa, 2012 in particular sought to ‘re-sensualise’ architecture by discerning the emotional states that are involved in the embodied experience of space, in contrast to the hegemony of ‘retinal architecture’ in modern western culture. By championing the return to a sensory architecture, Pallasmaa referred to the muscular tensions through which we apprehend the built environment, and on which we project our movements through a process of ‘bodily identification’ with place. He stressed the role of proprioception in the experience of space and recognised the unconscious desire to defy gravity that architecture can elicit:

‘The sense of gravity is the essence of all architectonic structures and great architecture makes us conscious of gravity and earth. Architecture strengthens verticality of our experience of the world. At the same time that architecture makes us aware of the depth of earth, it makes us dream of levitation and flight.’ (Pallasmaa, 2006: 37).

Accordingly, the heightened consciousness of gravity that is produced by awe-inspiring architectures is the source of ‘memorable experiences’. What remains unaccounted in this theory is the realm of sensory experiences that, in the presence of spatial depths, can engender emotions of fear and anxiety as well as comfort and pleasure. Further insights from psychology shed light on the ‘thrill of transparency’ that makes glass floors so appealing – although not for everyone.

The movements registered by the sensorimotor nerves define our overall body schema and thereby underpin the ways we organise our actions in space. As we have seen, the design of glass floors is intended to amplify the spectacle of the view from above by engaging the user's sense of balance when confronted with the sight of heights. When standing in front of a glass platform, a moment of realisation occurs whereby the incongruity of the view provokes an intense kinaesthetic feeling: an instinctive response that calls to mind the early experiments with depth perception based on the ‘visual cliff’ (Gibson and Walk, 1960). The kinaesthetic faculty that makes us aware of our bodily position in space (i.e., the ‘eye-object distance’) triggers the subjective feeling of imbalance that may cause discomfort and dizziness in ‘height intolerant’ subjects. Elevated glass floors are among those ‘encounter spaces’ that cause a high perception of risk in acrophobic sufferers (Andrews, 2007). At the opposite end of the spectrum are ‘height tolerant’ individuals who cope well with spatial depth and, in some cases, find excitement and exhilaration in the experience of altitude (Salassa and Zapala, 2009). The thrill of transparency induced by glass floors is what drives height-seeking individuals to revel in the psychological sense of danger that keeps others at bay. For thrill seekers, the sixth façade therefore constitutes a playground for the experience of the sixth sense.

While the present essay does not claim to provide a systematic analysis, these observations begin to delineate the spatial and social context in which the experience of elevated glass floors is situated. The links between physical sensations and the emotions related to the experience of heights introduce a further degree of complexity due to their fundamentally subjective nature. Psychoanalytic research suggests that feelings of anxiety and pleasure associated with vertigo are interwoven, as bodily perceptions reflect – and reveal – our dynamic mental states (Quinodoz, 1997). The ways in which inner drives are channelled through actions and behaviours is bound to determine varying levels of height tolerance through an individual's life span. To consider emotions of pleasure and anxiety as inextricably bound up might therefore help us to understand the irregular and mutable occurrences of vertigo that often punctuate people's lives. These embodied experiences are, however, invariably situated within a material and social context: hence the importance of considering the wider conditions in which glass floors and the related ‘thrills of transparency’ are produced.

## 6 Experience as commodity: the economy of vertigo

The idea of memorable experience advocated by the proponents of an architecture of the senses is an unholy bedfellow with the coeval ‘experience economy’ theory. With this influential term, Pine and Gilmore, 1998, Pine and Gilmore, 1999 named a step change in ‘the progression of economic values’ whereby businesses seek to gain a competitive edge by staging experiences that are purportedly memorable:

‘While prior economic offerings – commodities, goods, and services – are external to the buyer, experiences are inherently personal, existing only in the mind of an individual who has been engaged on an emotional, physical, intellectual, or even spiritual level.’ (Pine and Gilmore, 1998: n. p.)

The production of commodified, and increasingly customised, experiences spread from the entertainment industry to other sectors such as travel and retail, and extended to architecture as well (Lonsway, 2009). The tourist industry was among the first to embrace this process. Already in the 1970s MacCannell (1999/1976: 21) noted in his semiotic analyisis of tourist attractions: ‘Increasingly, pure experience, which leaves no material trace, is manufactured and sold like a commodity.’ Subsequently, Bauman (1996) pointed out that tourists, unlike other social types of travellers, are mainly driven by ‘pull’ rather than ‘push’ factors. In other words, their mobility is governed by aims (‘in order to’) rather than causes (‘because of’):

‘the tourist is a conscious and systematic seeker of experience, of a new and different experience, of the experience of difference and novelty – as the joys of the familiar wear off and cease to allure. The tourists want to immerse themselves in a strange and bizarre element (a pleasant feeling, a tickling and rejuvenating feeling, like letting oneself be buffeted by sea waves) – on condition, though, that it will not stick to the skin and this can be shaken off whenever they wish.’ (Bauman, 1996: 29)

These ideas, which to a large extent are still valid today, shed further light onto the rise of glass floors as ‘experience design’ products. To contemplate a cityscape through a horizontal window-floor is in itself a ‘strange and bizarre’ attraction that draws scores of visitors to immersive viewing galleries. Skywalks arguably constitute typical examples of ‘tourist bubbles’ (Judd, 1999): self-contained and highly regulated spaces in which moments of leisure can be enjoyed within secluded and safe environments. Marking a shift from the traditional mechanisms of panoramic vision, these spaces presuppose an expanded function of the tourist gaze involving a multi-sensuous, kinaesthetic experience: in other words, they are stages for the performance of ‘embodied actions’ (Urry and Larsen, 2012: 190). These actions are almost invariably recorded on camera, as the act of photographing or filming one's body suspended over the void is a popular means of validating the memorable experience.

Undeterred by the Global Financial Crisis of the late noughties, or perhaps even spurred by it, the skyscraper business has continued to tap into the growing market of vertiginous experiences. The design of multi-storey viewing galleries on top of ‘supertall’ buildings such as London's Shard and, more recently, New York's One World Trade Center has led to considerations that ‘observation decks have become cash machines.’ (Brown, 2014) Vying to attract visitors, today's viewing galleries stage the embodied experience of urban heights as an increasingly immersive event. Whilst vision is still predominant, its mechanisms and practices are increasingly augmented through immersive spatial experiences that find a parallel in the surge of technologies such as Virtual Reality, 4D cinema, and the like.

A technological variation on the theme of the glass floor is shown by the ‘sky portal’ built into the observatory's floor at One World Trade Center, which opened to the public in 2015. This attraction consists of a round 14 ft-wide platform where visitors can stand and watch a live video image of the scene unfolding at street level. The thrill of the glass floor is simulated by a high-definition livestream: in lieu of real transparency, the screen technology reproduces views from above to be watched in a vicarious state of suspension. By replacing the direct sight of height with an immersive cinematic spectacle the ‘sky portal’ takes the suspension of disbelief to a new level: further evidence of how deeply architectures of vertigo are embedded in the structures of a buoyant experience economy. The pursuit of memorable experiences leads to the production of spaces that, while largely homogenised, at the same time boast their own distinctive features – or, in business parlance, ‘unique selling points’.

## 7 Conclusion

This study suggests that observation decks are increasingly conceived as spaces of visceral thrills. The vogue of high-level glass floors could easily be dismissed as a passing fad driven by the ‘form follows fun’ principle; and yet, upon closer scrutiny this phenomenon reveals deeper socio-spatial implications. When considered together, the cases discussed above show a consistent shift from the realm of architectures of vision towards what might be called *architectures of vertigo*. Although it probably remains that, while on high, ‘one can feel oneself cut off from the world and yet the owner of a world’ (Barthes, 1983/1964: 250), the panoramic view alone appears to be no longer adequate to meet the demands of urban observatories. These places reflect, and actively produce, a collective desire for vertiginous experience akin to the ‘voluptuous panic’ described by Caillois as *ilinx*.

The proliferation of skywalks and sundry glass platforms signals that, amidst a thriving experience economy, designers have been perfecting new ways of harnessing the user's sensory responses to spatial depth. It can be hypothesised that the viewing subject central to modern scopic regimes is being superseded by a sentient subject whose feelings are put through ever more intense psycho-physiological stimuli. This subject embodies a visual sensibility that is no longer predicated on processes of abstraction and cognition but involves an expanded, and increasingly immersive, field of sensory experience. Glass floors in particular offer a kinaesthetic experience of space that combines the thrill of altitude with the thrill of transparency. As we have seen, these platforms owe their appeal to a horizontal transparency that provokes the exhilarating feeling of hovering over urban space as if in a state of suspension.

Elevated glass floors reproduce the groundlessness of the present socio-economic condition and effectively reaffirm it in spatial form. By so doing, they partake in the ‘dreamworlds of neoliberalism’ (Davis and Monk, 2007) that are shaping the emotional landscapes of our cities. Indeed, the spaces described in this paper comply with the predominant subjectivity in which risks and insecurities are governed through environmental stimuli that discipline the activities of the body. They are aligned with a broad strand of architecture that, since the 1990s, has been designed primarily to stimulate ‘affects’ (Spencer, 2016): a ‘post-critical’ design approach that, by disengaging the user from rational and cognitive processes, bolsters up the dominant logic of neoliberalism. In a similar vein, skywalks are designed to embolden a dynamic and enterprising subject to enjoy a seemingly boundless degree of freedom, albeit an artificially staged one. This avid consumer of novel and ever-more thrilling experiences embodies the zeitgeist of our hyper-hedonistic age; that is, what psychoanalysts have identified as a social imperative of the present moment – ‘*you must enjoy!’* (Recalcati, 2012: 111). In staging a playground for thrilling experiences, skywalks join a plethora of other games of *ilinx* that defy the laws of gravity and thereby exorcise the existential fear of falling.

Thrill-seeking has been associated with those forms of ‘deep play’ (Ackerman, 1999) that entice people who yearn to escape the restraints of civilized life, particularly in big cities, to attain ecstatic and enlightening moments through adventurous exploits. Skywalks are arguably symptomatic of this wider psychosocial condition. The drive to challenge one's sense of balance is an innate form of human play, and there is something fascinating, as well as frightening, about the possibility of looking down through an elevated glass floor. These platforms hold the potential to reveal hitherto invisible spaces, to provoke an awareness of the vertical expansion of cities, and possibly reflections about its hubristic nature. However, their main social function seems to be an altogether different one. Far from inviting a reflexive aesthetic experience, the viewing galleries that draw visitors to ‘walk on air’ operate as tourist bubbles in which the encounter with the abyss underneath our feet is reduced to a themed spectacle. As the sixth façade becomes the stage of memorable experiences, the hyper-secure conditions in which these experiences take place ensure that our perception of verticality is domesticated and normalised within highly-controlled spaces. This phenomenon offers insights into the increasingly vertiginous environments of contemporary cities and provokes reflections about the social and spatial conditions they reflect and in turn produce.

## Footnotes

1 In 2012 the newest ‘tallest tower in the world’ – Skytree Tokyo – was built in the Japanese capital. The broadcasting tower reaches an overall height of 634 m at the summit, with public observatories at 350 m and 450 m; it includes a spiralling *skywalk* and an observation deck partly lined with glass floors.

2 Other notable examples include the Cabo Girão Skywalk, built on a cliff of Madeira (2012); the ‘Step into the Void’ skywalk at Aiguille du Midi, on the Mont Blanc, branded as ‘the highest attraction in Europe’ (2013); and the glass-bottomed bridge over the Zhangjiajie Grand Canyon in China's Hunan province (2016), currently the longest in the world (375 m).
